# Use of 3D-CT-derived psoas major muscle volume in defining sarcopenia in colorectal cancer

**DOI:** 10.1186/s12885-024-12524-y

**Published:** 2024-06-18

**Authors:** Makoto Takahashi, Kazuhiro Sakamoto, Yosuke Kogure, Shuko Nojiri, Yuki Tsuchiya, Kumpei Honjo, Masaya Kawai, Shun Ishiyama, Kiichi Sugimoto, Kunihiko Nagakari, Yuichi Tomiki

**Affiliations:** 1 Department of Coloproctological Surgery, Faculty of Medicine, Juntendo University, Tokyo, Japan; 2 Department of Radiological Technology, Juntendo University Hospital, Tokyo, Japan; 3 Medical Technology Innovation Center, Juntendo University, Tokyo, Japan

**Keywords:** Sarcopenia, Colorectal cancer, Psoas major muscle volume, 3-dimensional computed tomography

## Abstract

**Background:**

Sarcopenia is characterized by reduced skeletal muscle volume and is a condition that is prevalent among elderly patients and associated with poor prognosis as a comorbidity in malignancies. Given the aging population over 80 years old in Japan, an understanding of malignancies, including colorectal cancer (CRC), complicated by sarcopenia is increasingly important. Therefore, the focus of this study is on a novel and practical diagnostic approach of assessment of psoas major muscle volume (PV) using 3-dimensional computed tomography (3D-CT) in diagnosis of sarcopenia in patients with CRC.

**Methods:**

The subjects were 150 patients aged ≥ 80 years with CRC who underwent primary tumor resection at Juntendo University Hospital between 2004 and 2017. 3D-CT measurement of PV and conventional CT measurement of the psoas major muscle cross-sectional area (PA) were used to identify sarcopenia (group S) and non-sarcopenia (group nS) cases. Clinicopathological characteristics, operative results, postoperative complications, and prognosis were compared between these groups.

**Results:**

The S:nS ratios were 15:135 for the PV method and 52:98 for the PA method. There was a strong positive correlation (*r* = 0.66, *p* < 0.01) between PVI (psoas major muscle volume index) and PAI (psoas major muscle cross-sectional area index), which were calculated by dividing PV or PA by the square of height. Surgical results and postoperative complications did not differ significantly in the S and nS groups defined using each method. Overall survival was worse in group S compared to group nS identified by PV (*p* < 0.01), but not significantly different in groups S and nS identified by PA (*p* = 0.77). A Cox proportional hazards model for OS identified group S by PV as an independent predictor of a poor prognosis (*p* < 0.05), whereas group S by PA was not a predictor of prognosis (*p* = 0.60).

**Conclusions:**

The PV method for identifying sarcopenia in elderly patients with CRC is more practical and sensitive for prediction of a poor prognosis compared to the conventional method.

## Background

Sarcopenia is defined as a reduction in skeletal muscle mass and is commonly observed in the elderly [1]. The prevalence of sarcopenia varies widely according to reports, ranging from 18.5% to 83.0% [2]. The prevalence of sarcopenia in solid malignant tumors is reported to be 35.3% in meta-analyses [3]. The reason for our focus on sarcopenia in this study is its significant utility in predicting the prognosis of many cancer patients. Specifically, in patients with various malignancies, sarcopenia has been identified as a highly relevant factor adversely affecting the treatment response to chemotherapy, increasing treatment related toxicity, and significantly worsening overall survival (OS) and disease-free survival (DFS), as reported by Alexey S in multiple studies [3–5]. Moreover, when limited to colorectal cancer (CRC), sarcopenia is associated with increased postoperative complications, prolonged hospital stay, deteriorated OS, DFS, and cancer-specific survival (CSS) [6]. Notably, it has been shown to worsen OS, particularly in patients undergoing first-line chemotherapy [7]. Therefore, assessing the presence of sarcopenia before initiating various treatments in cancer patients is of paramount importance, given its potent role as a prognostic factor for malignant tumors. On the other hand, recent data on site-specific cancer incidence in Japan has shown that the highest rate occurs in colorectal cancer (CRC), followed by lung cancer and gastric cancer [8]. Japan also has a growing elderly population, which has become a social problem, and it is reasonable to assume that a certain proportion of elderly patients with CRC will have sarcopenia. Thus, we focused our attention on sarcopenia in older patients with CRC in this study.

Therefore, sarcopenia is oncologically significant, emphasizing the necessity for a more accurate diagnosis of its presence or absence. One difficulty with sarcopenia is that there is no global standardized method for diagnosis. Various approaches have been used, including measurement of the psoas major muscle cross-sectional area (PA) at the L3 vertebral level, most commonly using computed tomography (CT), but also with magnetic resonance imaging (MRI), dual-energy X-ray absorptiometry (DXA), bioelectrical impedance analysis (BIA), and ultrasonography. A review by Vergara-Fernandez et al. in 2020 found that all 17 recent publications used the PA method with CT [9]. However, recently, Hirayama introduced a new approach that also uses CT, but focuses on the psoas major muscle volume (PV), which has proven to be a more sensitive reflection of skeletal muscle in the elderly population [10]. Furthermore, this approach has been shown to be closely associated with sarcopenia. The innovative aspect of this method lies in its ability to calculate PV swiftly and effortlessly, without the need for additional CT scans. Instead, the method harnesses CT data routinely collected during preoperative examinations for CRC, and thus, offers a remarkably simple and rapid assessment.

## Methods

### Aim

The aim of the study was to compare measurement of PV using 3-dimensional computed tomography (3D-CT) with the conventional method of measuring PA at L3 for identifying sarcopenia in elderly patients with CRC in the same population. Differences in patient characteristics, surgical outcomes, postoperative complications, and prognosis were examined in sarcopenia (group S) and non-sarcopenia (group nS) cases identified using each method.

### Participants

The study included a total number of 150 patients aged ≥ 80 years with Stage I-IV colorectal cancer who underwent primary tumor resection between 2004 and 2017 at Juntendo University Hospital in Japan [11]. Surgical procedures included open and laparoscopic surgery. All patients were performed conventional CT preoperatively, and we focused on patients for whom preoperative CT data could be imported into a 3D-CT workstation. Cases with duplicate malignancies, multiple cancers, and those requiring multi-organ resection were excluded. Cases with stoma creation only were excluded because primary tumor remains. This research was conducted in accordance with the principles established by the Declaration of Helsinki and approved by Institutional Review Board of Juntendo University (approval #19–243: date 29-May-2020). Institutional Review Board of Juntendo University waived the need for informed consent due to the nature of this retrospective and non-interventional study.

### Processes

**Fig. 1:**
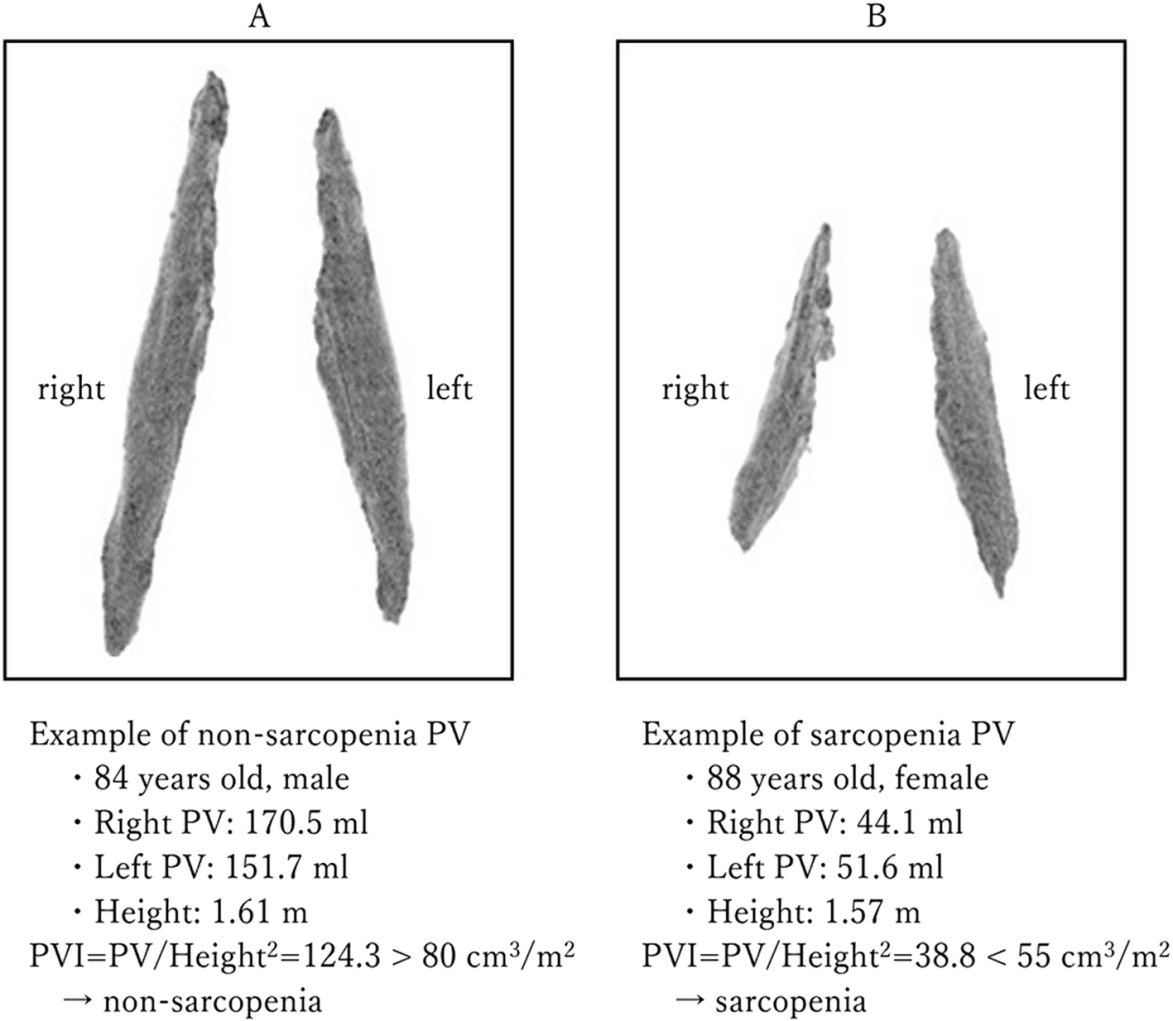
Three-dimensional view of the psoas major muscle volume (PV) using a 3D-CT workstation (ziostation2™, ziosoft, Tokyo, Japan). **A** Non-sarcopenia case. **B** Sarcopenia case

For diagnosis of sarcopenia, preoperative CT data were loaded on a 3D-CT workstation (ziostation2™, ziosoft, Tokyo, Japan). With a single click, the software automatically identified the psoas major muscle, generated a 3D image of the muscle, and calculated and displayed the volume of the muscle (PV). PV was divided by the square of the patient's height to obtain the psoas volume index (PVI). These indices were used to separate the data into two groups based on a certain cutoff value to identify cases with sarcopenia. The cutoff values were established by Hirayama for elderly Japanese patients, as PVI < 80 cm^3^/m^2^ for males and < 55 cm^3^/m^2^ for females [10]. 3D images of psoas muscles created by the software and examples of volumes are shown in Fig. 1. The figure depicts 3D representations of the left and right psoas muscles, along with the respective volumes used to calculate PVI. In Fig. 1A, PVI is 124.3 cm^3^/m^2^ and the male case is classified as non-sarcopenia (i.e., PVI > 80 cm^3^/m^2^). Conversely, in Fig. 1B, PVI is 38.8 cm^3^/m^2^ and the female case is classified as sarcopenia (i.e., PVI < 55 cm^3^/m^2^). The conventional method uses determination of PA at L3 on CT. PAI (psoas major muscle cross-sectional area index) is defined as the sum of the left and right PA divided by the square of height. Sarcopenia is then defined as PAI ≤ 6.0 cm^2^/m^2^ in males and ≤ 3.4 cm^2^/m^2^ in females. These cutoffs are established from data for Japanese patients across multiple facilities, based on criteria for diagnosis of sarcopenia proposed by The Japan Society of Hepatology, second edition [12]. This is a simplified method that does not require muscle mass measurement software. We note that the cutoff values may be subject to change based on further studies [12].

### Statistical analysis

Statistical analysis was conducted using JMP® v.11 (SAS Institute, Cary, NC, USA). Continuous variables were assessed by Wilcoxon and Kruskal–Wallis tests, while categorical variables were analyzed by Pearson χ^2^ test and Fisher exact test. Correlation between two variables was examined using the Pearson product moment correlation coefficient:* r*. The interpretation based on the absolute value of *r* was: | *r* |= 1.0 to 0.7: significant strong correlation; | *r* |= 0.7 to 0.4: strong correlation; | *r* |= 0.4 to 0.2: moderate correlation; | *r* |= 0.2 to 0.0: little to no correlation. Survival curves were generated using the Kaplan–Meier method. Multivariate analysis of survival rates was performed using a Cox proportional hazards model. Statistical significance was defined as *p* < 0.05.

## Results

The median age of the patients was 83 years, with a male: female ratio of 85:65. The median body mass index (BMI) was 22.5 kg/m^2^. The Glasgow Prognostic Score (GPS) was mainly 0, followed by 1 and 2. The American Society of Anesthesiologists (ASA) class was ASA 2 in most cases, with ASA 1 and 3 being less common. Individuals with relatively severe comorbid conditions such as heart, lung or kidney disease, hypertension, diabetes, collagen disease, and other conditions were categorized as ASA 3. A history of abdominal surgery was identified in 38.7% of cases. For the tumor location, the cecum, ascending colon, and transverse colon were defined as the right-sided colon, and the descending colon, sigmoid colon, rectosigmoid and rectum as the left-sided colon. The tumor location was evenly distributed between the right and left sides (75 cases each). TNM staging was 1 to 3 in most cases, with 9 cases at stage 4 [11]. Among surgical procedures, laparoscopic surgery was more common than open surgery (112 vs. 38 cases) (Table 1).

**Table 1:** Patient characteristics

Factor	*n* = 150
Median age (years old)	83 (80–97)
Sex (male/female)	85/65
BMI (kg/m^2^)	22.5 (14.8–32.5)
PVI (cm^3^/m^2^)	93.8 (38.8–184.4)
PAI (cm^2^/m^2^)	5.6 (1.3–12.0)
GPS (0/1/2)	97/25/24
ASA (1/2/3)	10/119/21
Previous abdominal surgery	58 (38.7%)
Tumor location (Right/Left)	75/75
Stage (0/1/2/3/4)	1/37/60/43/9
Laparoscopic / Open surgery	112/38

**Fig. 2:**
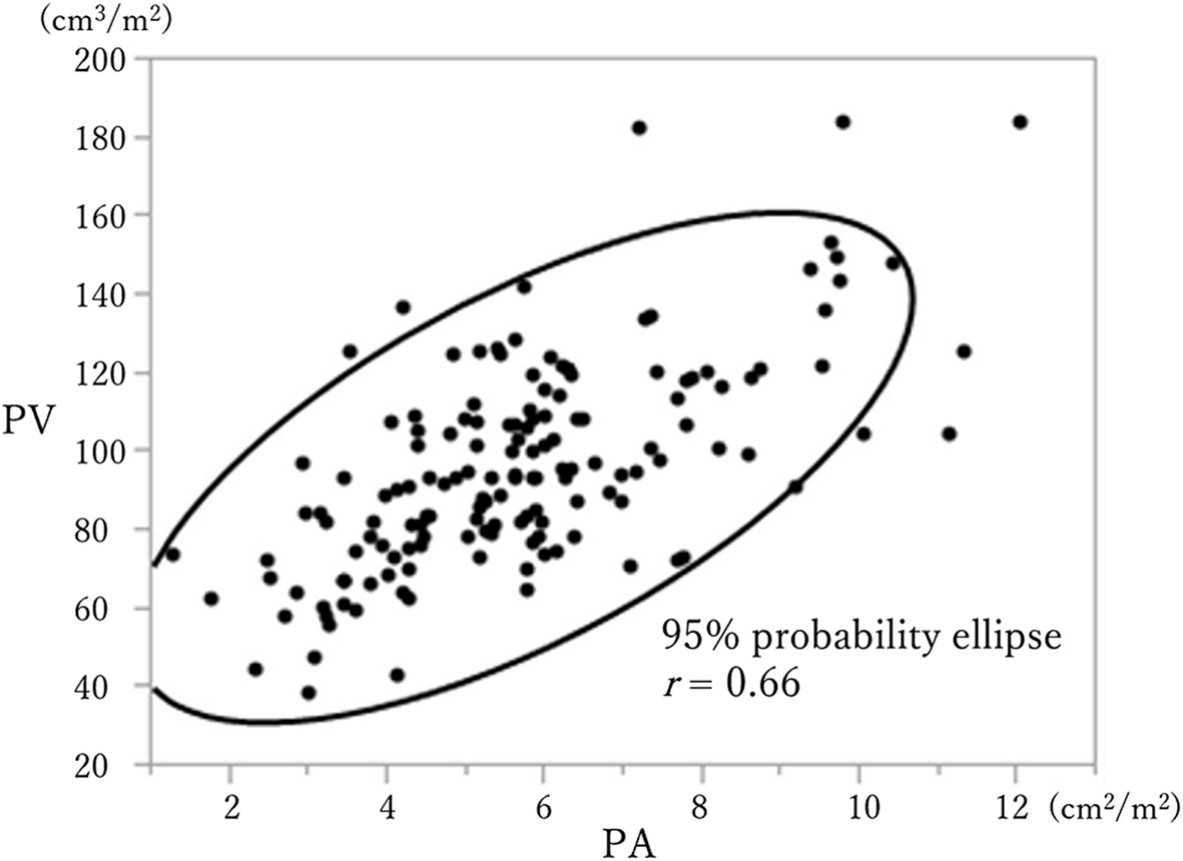
Scatter plots of PVI and PAI for investigating the correlation between PV and PA, with the 95% probability ellipse. The Pearson product moment correlation coefficient (*r*) was 0.66, indicating a strong positive correlation (*p* < 0.01)

In classifications using PV and PA, group S included 15 cases (10.0%) and 56 cases (37.3%), respectively. The correlation between PV and PA results was investigated using PVI and PAI. Histograms showed that both PVI and PAI followed normal distributions (data not shown). The Pearson product moment correlation coefficient (*r*) of a scatter plot for PVI vs. PVA was 0.66 (*p* < 0.01), indicating a strong correlation between the two variables (Fig. 2).

Next, we compared patient characteristics, surgical outcomes, postoperative complications, and prognosis in a comparative analysis using the PV and PA methods for sarcopenia assessment. There were no significant differences in patient characteristics between groups S and nS based on the PV method, but group S based on the PA method had a significantly higher proportion of males (*p* < 0.01) and significantly lower BMI (*p* = 0.03) (Table 2). Up to this point, we have considered both the curative and palliative settings (*n* = 150). However, in the subsequent analysis, we focused solely on the curative setting (*n* = 131) due to the potential impact of TNM Stage 4 cases, i.e., palliative setting, on surgical outcomes and prognosis. In groups S and nS based on the PV and PA methods, there were no significant differences in surgical outcomes (Table 3) and postoperative complications defined as Clavien-Dindo grade 2 or higher [13] (Table 4).

**Table 2:** Univariate analysis of characteristics of patients with (group S) and without (group nS) sarcopenia based on the PV and PA methods

Method	PV			PA		
Group	Group nS *n* = 135	Group S *n* = 15	*p* value	Group nS *n* = 94	Group S *n* = 56	*p* value
Age (years old)	83	85	0.09	83	82.5	0.49
Sex (male/female)	74/61	11/4	0.27	43/51	42/14	< 0.01
BMI (kg/m^2^)	22.9	20.5	0.06	23.0	21.5	0.03
GPS (0/1/2)	91/20/20	6/5/4	0.07	59/15/16	38/10/8	0.86
ASA (1/2/3)	10/106/19	0/13/2	0.54	4/77/13	6/42/8	0.30
Previous abdominal surgery	53 (39.3%)	5 (33.3%)	0.78	35 (37.2%)	23 (41.1%)	0.73
Tumor location (Right/Left)	70/65	5/10	0.28	44/50	31/25	0.31
Stage (0/1/2/3/4)	1/33/53/41/7	0/4/7/2/2	0.53	0/25/37/25/7	1/12/23/18/2	0.50
Laparoscopic / Open surgery	102/33	10/5	0.53	68/26	44/12	0.44

**Table 3:** Surgical outcomes for patients with (group S) and without (group nS) sarcopenia based on the PV and PA methods

Method	PV			PA		
Group	Group nS *n* = 128	Group S *n* = 13	*p* value	Group nS *n* = 87	Group S *n* = 54	*p* value
Operative time (minutes)	246	207	0.08	245	247.5	0.75
Blood loss (ml)	30	20	0.16	30	30	0.91
LN dissection level (1/2/3)	8/39/81	0/6/7	0.39	4/26/57	4/19/31	0.57
LN harvest number	17	14	0.62	17	14	0.36
Days to solid diet (days)	4	4	0.95	4	4	0.76
Postoperative complications	47 (36.7%)	3 (23.1%)	0.38	33 (37.9%)	17 (31.5%)	0.44
Length of stay (days)	11	12	0.87	11	12	0.30

**Table 4:** Details of postoperative complications in patients with (group S) and without (group nS) sarcopenia based on the PV and PA methods

Method	PV			PA		
Group	Group nS *n* = 128	Group S *n* = 13	*p* value	Group nS *n* = 87	Group S *n* = 54	*p* value
Delirium	22 (17.2%)	2 (15.4%)	0.60	16 (18.4%)	8 (14.8%)	0.58
Urinary disturbance	10 (7.8%)	0 (0.0%)	0.60	7 (8.1%)	3 (5.6%)	0.74
Ileus	7 (5.5%)	0 (0.0%)	1.00	2 (2.3%)	5 (9.3%)	0.11
Anastomotic leakage	3 (2.3%)	0 (0.0%)	1.00	1 (1.2%)	2 (3.7%)	0.56
Superficial SSI	3 (2.3%)	0 (0.0%)	1.00	3 (3.5%)	0 (0.0%)	0.29
Enteritis	3 (2.3%)	0 (0.0%)	1.00	2 (2.3%)	1 (1.9%)	1.00
Urinary infection	2 (1.6%)	0 (0.0%)	1.00	0 (0.0%)	2 (3.7%)	0.15

*SSI* surgical site infection

**Fig. 3:**
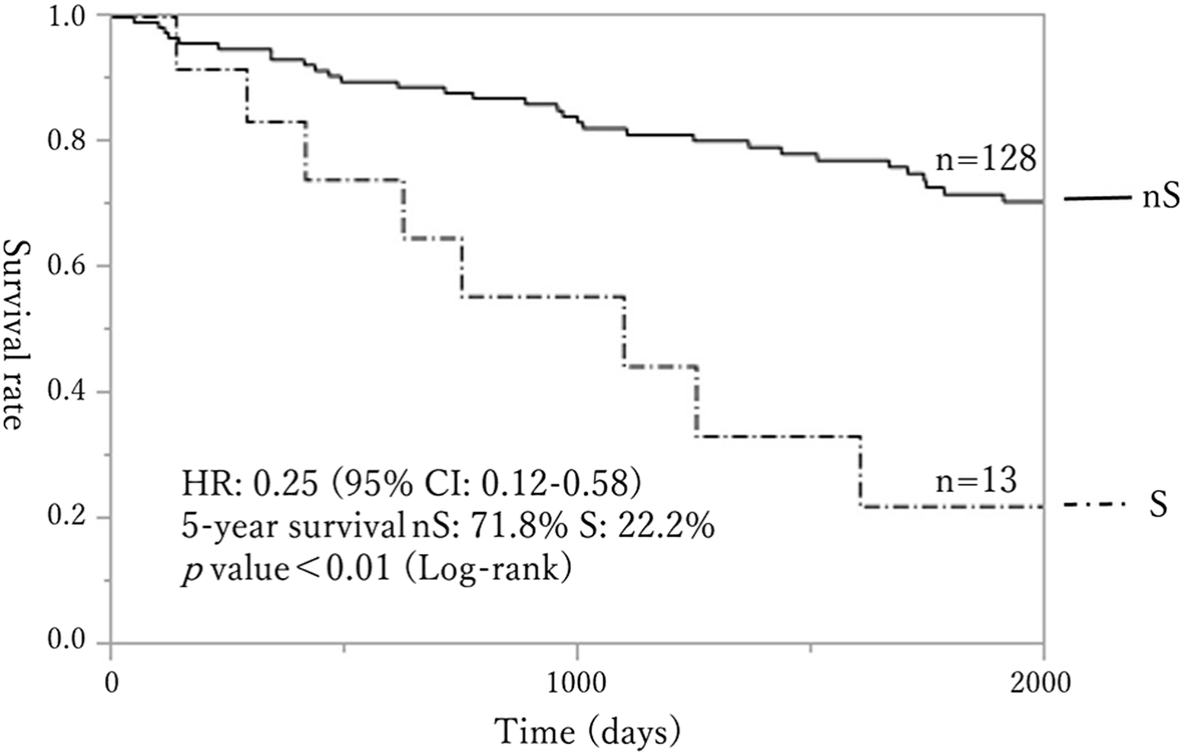
Survival curves for the sarcopenia (S) and non-sarcopenia (nS) groups defined using the PV method. Group S had a significantly poorer prognosis (HR: 0.25, 95% CI: 0.12–0.58, 5-year survival: S 22.2% vs. nS 71.8%, *p* < 0.01 by log-rank test)

**Fig. 4:**
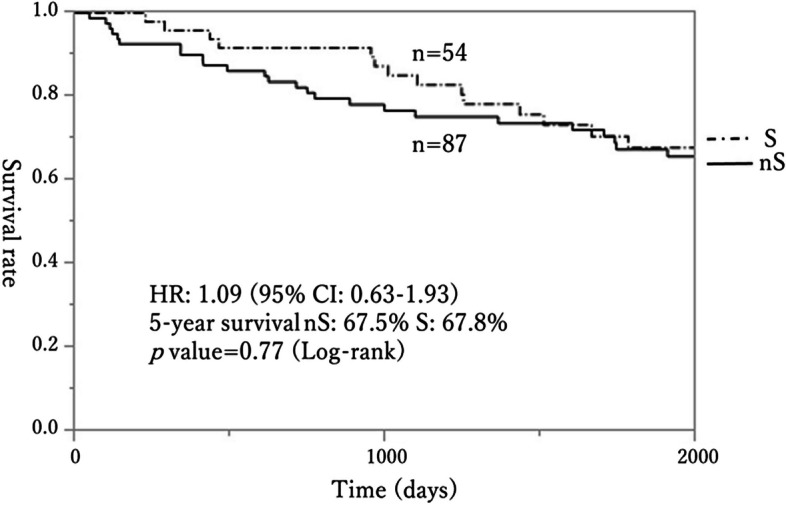
Survival curves for the sarcopenia (S) and non-sarcopenia (nS) groups defined using the PA method. The two curves are close to each other and there was no significant difference (*p* = 0.77 by log-rank test)

Group S defined by the PV method had a significantly worse OS curve compared to group nS (HR 0.25, 95% CI: 0.12–0.58, Fig. 3). The 5-year OS rates were 22% for group S and 71.8% for group nS (*p* < 0.01, log-rank test). However, using the PA method, there was no difference in OS curves between the two groups (*p* = 0.77; log-rank test, Fig. 4). Cox proportional hazards models were built to identify factors associated with worsened OS (Table 5). Confounding factors included age, gender, sarcopenia, GPS, ASA, tumor location, TNM Stage, and Japanese classification of lymph node (LN) dissection [14, 15]. BMI was not included as a confounding factor because several studies have reported an association between BMI and sarcopenia, with lower BMI associated with a higher risk of sarcopenia [16]; therefore, we chose to include sarcopenia and did not include BMI. Also, as mentioned above, there was a robust correlation (*r* = 0.66) between PVI and PAI. Thus, instead of performing multivariate analysis with PV and PA as concurrent confounding factors for sarcopenia, two separate Cox proportional hazards models were created: one for PV and one for PA (Table 5). In the PV model, sarcopenia was identified as an independent predictor of a poor prognosis (HR: 2.55, 95% CI: 1.02–5.68, *p* < 0.05, Table 5). In contrast, in the PA model, sarcopenia was not a predictor of a poor prognosis (*p* = 0.60), but age (HR: 1.10, 95% CI: 1.01–1.19, *p* = 0.03) and sex (HR: 2.00, 95% CI: 1.07–3.87, *p* = 0.03) were identified as significant predictors for a poor prognosis (Table 5).

**Table 5:** Cox proportional hazards models for prognosis based on classification of sarcopenia using the PV and PA methods (asterisk (*) represents 95% CI)

	PV Method		PA Method	
Factor	Hazard Ratio	*p* value	Hazard Ratio	*p* value
Age		0.08	1.10 (1.01–1.19) *	0.03
Sex		0.07	2.00 (1.07–3.87) *	0.03
Sarcopenia	2.55 (1.02–5.68) *	< 0.05		0.60
GPS (0/1,2)		0.37		0.42
ASA (1,2/3)		0.06		0.09
Tumor location (Right/ Left)		0.09		0.08
Stage (1/2, 3)		0.97		0.81
LN D1, D2/D3		0.71		0.62

## Discussion

The average life expectancy in Japan is the second-highest worldwide for men, at 81.5 years, and the highest for women, at 86.9 years [17]. Furthermore, Japan has one of the highest proportions of elderly citizens (age ≥ 65), with a rate of 28.9% [18]. Therefore, the probable high rate of individuals with sarcopenia in Japan suggests that research on this condition is of utmost importance. Sarcopenia is broadly attributed to two primary causes: age-related physiological changes leading to a reduction in skeletal muscle mass and secondary changes resulting from chronic conditions such as chronic obstructive pulmonary disease (COPD), acquired immunodeficiency syndrome (AIDS), and various cancers, including CRC. Notably, the presence of sarcopenia in cancer patients is associated with worse prognoses compared to those without sarcopenia [19]. Indeed, sarcopenia is recognized as an adverse prognostic factor for many malignant tumors, with hazard ratios ranging from 1.11 to 2.12 [20]. Additionally, sarcopenia has a negative impact on outcomes and reduces quality of life (QOL), increases susceptibility to depression, and has other adverse effects [21].

Cancer patients who overcome sarcopenia following surgery tend to have improved outcomes compared to those who continue to be affected by sarcopenia [22]. These findings emphasize the importance of identifying sarcopenia in cancer patients. Consequently, our focus in this study was to explore how to identify sarcopenia in CRC cases. There are various methods for diagnosis of sarcopenia, including CT, MRI, ultrasound, DXA, and BIA [23–25]. Each method has its benefits and drawbacks, including the need for specialized equipment, radiation exposure, ease of use for medical staff, and patient convenience. Among these methods, CT has been widely used in the past few years for measurement of PA at the L3 vertebral level. In fact, there are numerous meta-analyses on the impact of sarcopenia on prognosis; however, most of them diagnose sarcopenia using the cross-sectional area of the major psoas muscle at the L3 level, namely PA method [3–7]. There are very few studies that have compared and examined the same CRC cases using both the potentially more accurate PV method and the conventional PA method, which represents two different approaches to extracting sarcopenia. In CRC, there are reports on use of PV and PA for post-chemoradiotherapy rectal cancer and postoperative complications [26–30], but we could not find any studies on long-term prognosis and 5-year survival rate in elderly patients with CRC. In this regard, our study is novel in addressing these perspectives. In previous reports, Horie et al. have suggested that evaluating PV may have higher reliability than PA since it involves measuring a broader range of the muscle, thereby reducing errors [26]. In PA measurement, the measured height may not correspond to the maximum area, and significant variations can occur depending on the level of measurement [27, 28]. So et al. argued that 3D assessments in patients with hip fractures are more accurate for evaluating muscle mass compared to 2D methods [29]. On the other hand, one report suggested that both PA and PV are effective [30]. We considered how these approaches may be applicable in elderly individuals with spinal curvature, and we concluded that volume assessment (PV) would allow for more accurate diagnosis of skeletal muscle mass (i.e., sarcopenia) compared to PA. We also note that preoperative CT scans for CRC were performed in all cases, and the data were simply imported into a 3D-CT workstation, on which PV could be automatically determined. This process is convenient and efficient for both medical staff and patients, and it also reduces radiation exposure.

In comparing the PV and PA methods for diagnosis of sarcopenia in the same 150 cases, one striking observation was the notable difference in the proportion of sarcopenia cases between the two methods: 10.0% with PV vs. 34.7% with PA. Generally, the rate of sarcopenia in older adults with various types of cancer, including CRC, ranges from 18.5% to 83.0% [2], making our PV data appear notably low. One of the reasons for this discrepancy may be the newness of the PV method, with definitive cutoff values yet to be established. Psoas muscle volumes are influenced by factors such as height, weight, and ethnicity, requiring the need for normalization by dividing by height. However, there are methods involving height squared and cubed for this purpose, and as a result, cutoff values remain uncertain. It is possible that the cutoff used in this study was somewhat stringent [10, 26, 27]. We also found a strong correlation between PVI and PAI (*r* = 0.66), as illustrated in Fig. 2. This was expected since both methods diagnose sarcopenia, implying that they both should identify the same group of patients with sarcopenia. In fact, Womer et al. have reported that PA and PV are both important parameters [30].

The question arises with regard to which method is more appropriate. To determine this, we assessed whether cases identified as having sarcopenia by both methods had the expected worse prognosis, using statistical analyses, survival curves (Figs. 3 and 4) and Cox proportional hazards models (Table 5). The results clearly showed that sarcopenia cases identified by the PV method had a significantly worse prognosis than those identified by the PA method. In Cox proportional hazard analysis, the adverse prognostic factors were sarcopenia with the PV method, and age and gender with the PA method. It is reasonable that older age is associated with a shorter lifespan and overall functional decline, and age is viewed as an adverse prognostic factor in this context. Concerning gender, females with CRC are generally considered to have a poorer prognosis than males. This is partially attributable to the higher incidence of right-sided CRC in females, which, compared to left-sided CRC, often exhibits features such as microsatellite instability (MSI), CpG island methylator phenotype (CIMP), and BRAF mutations, which contribute to a poorer prognosis [31]. For ASA and tumor location, the *p*-values were relatively small (< 0.10) with the PV and PA methods, whereas other factors (GPS, TNM Stage, LN dissection) had relatively large *p*-values in multivariate analysis. These factors are commonly associated with adverse prognosis, but were relatively unimportant compared to sarcopenia, age, and sex in this study. Thus, while TNM Stage and LN dissection are generally expected to influence CRC prognosis, under the specific conditions in this study (elderly patients with CRC aged 80 or above, excluding TNM Stage 4) the influence of sarcopenia, age, and gender may have masked the effects of TNM Stage and LN dissection. These findings suggest that the PV method is superior to the PA method for identification of sarcopenia.

It is also important to understand the mechanisms underlying the well-established effects of sarcopenia in worsening the prognosis of malignancies [19]. One explanation is that sarcopenia is related to immunological deterioration and aging, which in turn may promote cancer progression and increase systemic inflammation [26, 32]. It is also important to recognize that skeletal muscles support movement and support, and also serve as secretory organs. Skeletal muscles produce and release hundreds of peptides and proteins, which are referred to as myokines and cytokines. Among these, myostatin is associated with transforming growth factor-β, interleukin-15, NK cells, CD3, and CD8 T cells, all of which are linked to tumor progression. A decline in skeletal muscle mass can disrupt the balance of cytokines, potentially contributing to cancer progression and recurrence [27, 33–35]. For these reasons, the coexistence of sarcopenia in cancer has been associated with progression and a worsened prognosis, which includes a poorer survival curve.

In recent years, there have been reports not only on the volume and the area of skeletal muscle but also on the skeletal muscle density, with individuals having low skeletal muscle density often referred to as having myosteatosis [36–40]. Moreover, muscle density can also be measured using CT, similar to PA and PV. It is noted that patients with such conditions, when diagnosed with cancer, are prone to worsened OS and DFS and an increased incidence of postoperative complications. This implies that, beyond muscle volume, muscle density is considered indispensable for predicting the prognosis of cancer treatments. From this perspective, routine physical activity, including aerobic exercise, and activities that engage muscles are deemed crucial. While effective treatments for sarcopenia are deemed non-existent [41], considering from the perspective of muscle density, preoperative rehabilitation and regular muscle engagement may be potentially effective.

This study has several limitations. First, it is a single-center study with a relatively small number of cases. Additionally, older cases were included for which calculation of PV using the workstation software was not possible due to limitations in CT data handling. Second, the study is retrospective. Third, it is uncertain whether the cutoff values for PVI and PAI were appropriate. As mentioned above, cutoff values in this field are not universally established, both internationally and domestically, which is a challenge for future research. However, the cutoffs used in the study were chosen carefully, as they are tailored for the relatively smaller body size of the Japanese population, with a particular focus on the elderly, in contrast to the larger body sizes found in other countries. If the cutoff value for PA was set more strictly, it could be as important a factor as PV, and one study has found that both PA and PV are important [30]. There is also an issue regarding the diagnostic criteria for sarcopenia. The diagnostic approach recommended in Asia, including Japan, is comprehensive, assessing both loss of skeletal muscle mass and of muscle strength and physical performance, including handgrip strength, 6-m walk, the Short Physical Performance Battery (SPPB), and the 5-time chair stand test [42]. In Europe, there is emphasis on the importance of "low strength" in the criteria [43]. In this retrospective study, collecting data beyond skeletal muscle CT scans was challenging. As in many studies, we focused on skeletal muscle mass, which is the fundamental and critical component of sarcopenia criteria, but this limitation warrants acknowledgment. Moving forward, it will be important to amass prospective data encompassing both muscle mass and muscle strength and physical performance, with significant implications for clinical applications.

## Conclusions

In elderly CRC patients aged 80 and above, the PV method, as compared to the PA method, easily and more accurately identified a sarcopenia subgroup with a poorer prognosis. Therefore, the PV method appears to be effective for precise sarcopenia diagnosis.

## Data Availability

The data that support this study are available from the corresponding author upon reasonable request.

## Abbreviations

CRC: Colorectal cancer
3D: 3-Dimensional
CT: Computed tomography
PV: Psoas major muscle volume
PA: Psoas major muscle cross-sectional area
PVI: Psoas major muscle volume index
PAI: Psoas major muscle cross-sectional area index
OS: Overall survival
MRI: Magnetic resonance imaging
DXA: Dual-energy X-ray absorptiometry
BIA: Bioelectrical impedance analysis
BMI: Body mass index
GPS: Glasgow Prognostic Score
ASA: American Society of Anesthesiologists
COPD: Chronic obstructive pulmonary disease
AIDS: Acquired immunodeficiency syndrome
QOL: Quality of life
MSI: Microsatellite instability
CIMP: CpG island methylator phenotype
TGF: Transforming growth factor
IL: Interleukin
SPPB: Short Physical Performance Battery
